# Thermal History Effects on DSC Kinetic Analysis of DGEBA–DICY Epoxy Curing

**DOI:** 10.1021/acsomega.6c02244

**Published:** 2026-10-05

**Authors:** Paulo E. Ferreira, Baltus C. Bonse, João G. R. Poço

**Affiliations:** † 154620 Instituto de Pesquisas Tecnológicas do Estado de São Paulo, Departamento de Nanobiomanufatura, 05508-901, São Paulo, SP, Brazil; ‡ Centro Universitário FEI, Departamento de Engenharia Mecânica, 09850-901, São Bernardo Campo, SP, Brazil; § Centro Universitário FEI, Departamento de Engenharia Química, 09850-901, São Bernardo Campo, SP, Brazil

## Abstract

The curing behavior of diglycidyl ether of bisphenol A (DGEBA) epoxy resin with dicyandiamide (DICY) was evaluated using differential scanning calorimetry (DSC) and standard industrial kinetic methods. Melt-extruded powder samples were analyzed at heating rates of 5–20 °C min^–1^, under isothermal conditions from 180 to 210 °C, and through combined isothermal–dynamic experiments to assess residual curing. The Borchardt–Daniels method yielded apparent activation energies that depended on the heating rate, ranging from 177 ± 2 kJ/mol to 127 ± 4 kJ mol^–1^. The ± values correspond to the confidence limits reported by STARe for the numerical fit and should not be interpreted as measures of experimental reproducibility. A peak-based Kissinger analysis resulted in an apparent activation energy of about 108 kJ mol^–1^, whereas the isothermal Arrhenius analysis yielded approximately 84 kJ mol^–1^. Both values had relatively wide confidence intervals because of the limited number of experimental conditions. The nonextruded reference sample, analyzed at 10 °C min^–1^, showed a lower reaction enthalpy (89.45 versus 118.54 J g^–1^) and a higher peak temperature (234 versus 203 °C) than the extruded material. This suggests that melt processing changed the initial state of the formulation. However, the DSC results alone do not allow us to determine whether this was mainly due to better dispersion, partial dissolution of DICY, or some early-stage reaction during processing. The glass-transition temperature increased with conversion but remained well below the isothermal curing temperature, indicating that classical vitrification was not observed. The linear relationship between ln­(A) and *E*_a_ (R2 > 0.99) is interpreted as a correlation between the fitted Arrhenius parameters and may reflect both changes in the material and covariance between the parameters. Overall, the kinetic values obtained here should therefore be regarded as protocol-dependent phenomenological descriptors rather than intrinsic mechanistic constants.

## Graphical abstract

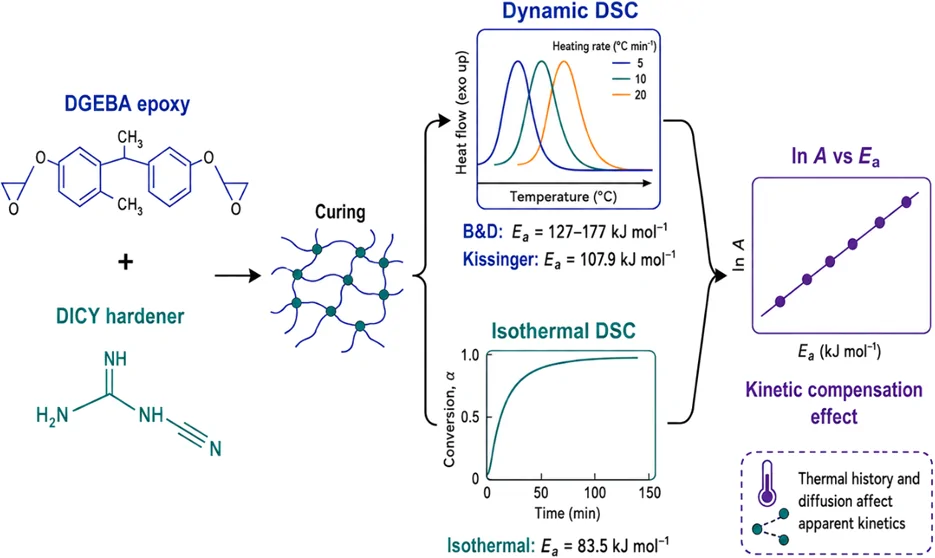

## 1 Introduction

Owing to their superior mechanical strength and dimensional stability, epoxy resins are the dominant matrix for polymer composites.^1–4^ However, the ultimate properties of these thermosets are governed by the curing parameters, including stoichiometry, temperature, and duration.^5^ Differential scanning calorimetry (DSC) is commonly employed to monitor the entire curing exotherm, enabling determination of heat flow profiles, in situ glass transition (*T*_g_), and optimal cure cycles.^6,7^

The DGEBA–DICY system is often used in high-performance products such as powder coatings and prepregs due to its unique latent-curing properties.^8^ Dicyandiamide (DICY) does not react at room temperature because of its high melting point of about 209 °C and its poor solubility in epoxy resins. As a result, the system has excellent shelf stability and can last 6 to 12 months.^9^ When heated, the terminal epoxide groups in diglycidyl ether of bisphenol A (DGEBA) react with the amine groups in DICY. Recent studies show that this reaction usually follows an autocatalytic kinetic model. First, hydroxyl groups formed during the ring-opening reaction make hydrogen bonds with unreacted epoxy groups, which helps the remaining amino groups react.^9–12^ Differential scanning calorimetry (DSC) often shows a sharp increase in reaction rate at a certain point.^10^

The cure of a diglycidyl ether of bisphenol A (DGEBA) epoxy with an amine hardener is highly exothermic and intrinsically complex. Initially, the amine attacks the epoxy ring, generating hydroxyl groups that subsequently catalyze further epoxy-opening reactions. Consequently, the curing process is typically autocatalytic and is frequently described using Kamal-type or Sesták–Berggren kinetic models rather than simple *n* ^th^-order approaches.^13–15^

Gelation and vitrification are two distinct transitions that can affect the curing of thermosets. Gelation marks the formation of an infinite network, resulting in the loss of macroscopic flow, while vitrification occurs when the conversion-dependent glass-transition temperature, *T*_g_(α), approaches the curing temperature. Both processes can lead to deviations from purely chemically controlled kinetics as network formation and reduced segmental mobility progressively limit molecular movement. However, restricted mobility alone should not be taken as evidence of vitrification unless the Tg­(α) criterion is also met.^16,17^

These multistage effects are critical for designing cure cycles, as they dictate the isothermal hold and ramp rates required for optimal composite processing. Because of this complexity, phenomenological kinetic models are typically fitted to DSC data. Techniques such as dynamic Kissinger analysis, Coats–Redfern plots, and isoconversional methods^2^ are often used to estimate the activation energy (*E*_a_) and pre-exponential factor (A). Simple *n* ^th^-order models alone often fail to capture real curing behavior, whereas two-parameter autocatalytic models can better reproduce observed kinetics. In addition, numerous DSC-based analysis methods (dynamic and isothermal) have been summarized for epoxy cures.^13,18–21^

Dicyandiamide (DICY) is a common epoxy hardener with very low solubility in the resin. The cure with DICY is negligible below ∼160–180 °C due to its low reactivity and phase separation at ambient temperatures. The DICY molecule (cyanoguanidine, C_2_H_4_N_4_) contains reactive primary and secondary amine groups as well as a nitrile group. During curing, DICY’s amine functions first add to the epoxy rings. This reaction is often catalyzed by the hydroxyls formed in the early stages. As the cure progresses, direct epoxy–epoxy reactions can also occur. Detailed mechanisms are illustrated by Flammersheim.^22^ Because DICY cures only at elevated temperatures, a higher cure temperature promotes its dissolution and reactivity, thereby accelerating the overall reaction^23–25^

In practice, additives and catalysts can significantly modify this curing kinetics. For example, adding a hyperbranched epoxy (HTDE) into DGEBA was found to accelerate the cure: the DSC exotherm shifts to a lower temperature, its peak sharpens, and the apparent activation energy decreases compared to neat DGEBA.^26^ Likewise, the choice of imidazole catalyst affects the cure profile: a primary imidazole (H-MI) produces the cure exotherm ∼10 °C lower than 1-methylimidazole (1-MI), corresponding to a slightly lower *E*_a_.^27,28^ These studies observed that the maximum cure rate occurred at roughly 30–40% conversion, which is characteristic of autocatalytic behavior. They also confirmed that vitrification occurs at high conversion, as evidenced by a steep drop-off in residual DSC heat and reaction rate near the polymer’s rising *T*_g_. Notably, these investigations reported a kinetic compensation effect: fitted values of ln *A* and *E*_a_ fall on a straight line, indicating a correlated change in the parameters. This compensation behavior has been documented in DSC cure kinetics, solid-state reactions, and heterogeneous catalysis.^29–33^

Although there is extensive research on epoxy curing kinetics, a key challenge remains: different kinetic methods often yield widely different activation energies for the same thermosetting system. This issue is especially noticeable in industrial latent-curing systems like DGEBA–DICY, where factors such as extrusion, partial dissolution, and vitrification may affect how the cure appears to behave.

While advanced kinetic models, such as the Kamal autocatalytic equation and the Sesták–Berggren formalism, are widely used for epoxy–amine systems, simpler industrial methods, such as Borchardt–Daniels analysis, remain common in quality control and process optimization. However, the drawbacks of using these simpler methods for latent-curing systems have not been adequately discussed in the literature.

This study critically compares the apparent kinetic parameters obtained from dynamic Borchardt–Daniels analysis, the peak-based Kissinger method, isothermal Arrhenius analysis, and combined residual-cure experiments for an industrial DGEBA–DICY powder formulation. The objective is to identify the extent to which the resulting parameters depend on the analytical protocol and to examine whether the archived DSC evidence is consistent with effects of melt processing, hardener state, network formation, and parameter covariance. The study does not attempt to assign the observed differences to a unique molecular mechanism.

## 2 Experimental Methods

The study utilized a solid DGEBA epoxy resin (Dow DER 664U) and a dicyandiamide curing agent (Huntsman Aradur 986). A stoichiometric mixture consisting of 95 wt % resin and 5 wt % curing agent was prepared. Approximately 950 g of DGEBA resin (Figure 1) and 50 g of DICY hardener (Figure 2) were premixed in a knife mill to ensure homogeneous powder distribution before extrusion.

**Figure 1:**
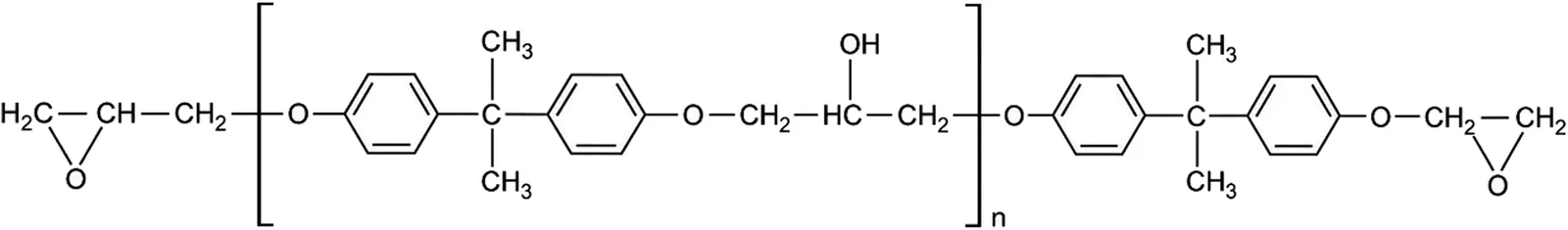
Structure of bisphenol A (DGEBA) epoxy.

**Figure 2:**
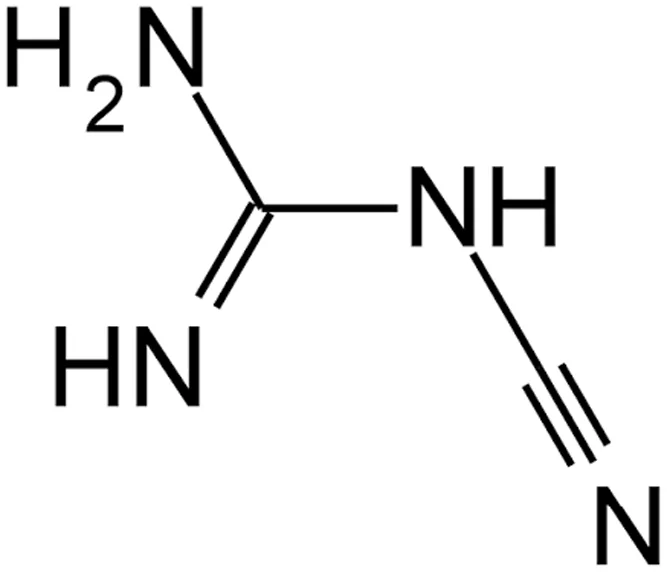
Structure of dicyandiamide (DICY) hardener

Subsequently, the blend was melt-processed in a Werner-Pfleiderer twin-screw extruder at 90 °C to simulate industrial powder-coating conditions. The extrusion process was conducted at a screw speed of 150 rpm and an estimated residence time of approximately 45–60 s. The temperature profile was maintained below the onset of bulk curing to minimize extensive cross-linking during processing. Nevertheless, the combined effects of temperature and shear may have modified DICY dispersion, dissolution state, and the initial extent of reaction. The resulting extrudate was immediately quenched on a chilled surface to arrest the reaction, then ground and sieved through a 112 μm mesh to yield the final powder. A small portion of the mechanically premixed DGEBA–DICY formulation was collected before extrusion, sieved, and analyzed by dynamic DSC at 10 °C min^–1^. This nonextruded material was used as a qualitative experimental reference for evaluating the influence of melt processing on the subsequent thermal response.

For calorimetry, samples were analyzed using a DSC on a Mettler Toledo 822e instrument equipped with an intracooler and a nitrogen purge. Nitrogen was used as purge gas at a nominal flow rate of 50 mL min^–1^, as recorded in the original STARe thermograms. Standard hermetically sealed aluminum pans were employed because of their high thermal conductivity and widespread use in epoxy DSC analysis, minimizing the thermal lag during the curing exotherm.

For each experiment, approximately 8–12 mg of extruded powder was weighed directly into the DSC pan immediately before analysis. Since the resin and curing agent had already been premixed and extruded, no additional mixing step was required prior to DSC measurements.

Dynamic DSC scans were performed from 25 to 300 °C at constant heating rates of 5, 10, 15, and 20 °C min^–1^.

Isothermal measurements^34^ were carried out at 180, 190, 200, and 210 °C using a Mettler Toledo DSC 822e equipped with an intracooler and a nitrogen purge. The DSC cell was preheated to the desired temperature, and the sample was added within about 8 s. Data collection continued until the curing process was fully complete. To ensure accuracy, temperature and enthalpy calibration checks were performed before the experiments using indium and lead standards, following established procedures.

Combined isothermal–dynamic residual-cure experiments were performed at 180 °C. Separate samples were held isothermally for 1, 2, 5, 7, 10, 12, or 15 min and were subsequently analyzed by a dynamic DSC scan at 10 °C min^–1^. The conversion reached during the isothermal pretreatment was calculated from the residual reaction enthalpy as α = 1−Δ*H*_res_/Δ*H*_0_, where Δ*H*_0_ is the reaction enthalpy of the untreated material measured under the same dynamic program. The Borchardt–Daniels calculation was then applied to each residual exotherm. Each hold time was represented by one sample; the different hold times therefore constitute a conversion series and not experimental replicates.

Kinetic analyses were carried out using the manufacturer’s STARe software. The curing conversion (α) was calculated from the ratio between the partial reaction enthalpy released at a given time and the total reaction enthalpy (Δ*H*_total_), according to α = Δ*H*_t_/Δ*H*_total_, where Δ*H*_t_ corresponds to the cumulative heat released during curing.

In all instances, the measured heat flow (d*H*/d*t*) was assumed to be directly proportional to the reaction rate (d*a*/d*t*) at any given conversion, according to d*a*/d*t* = (1/Δ*H*_total_) d*H*/d*t*, where *H* represents the reaction enthalpy measured by DSC and α is the degree of conversion.

The dynamic DSC data were analyzed using the Borchardt–Daniels *n* ^th^-order method. This model assumes that the curing reaction follows: d*a*/d*t* = *k*(1–*a*)*^n^*, where *k* is the kinetic constant and *n* is the apparent reaction order.

The temperature dependence of the kinetic constant was described by the Arrhenius equation: *k* = *A* exp­(−*E*_a_/*RT*), where *A* is the pre-exponential factor, *E*_a_ is the activation energy, *R* is the gas constant, and *T* is the absolute temperature.

To reduce the model dependence associated with the Borchardt–Daniels approach, the Kissinger peak-based multiple-heating-rate method was also applied using peak temperatures obtained at heating rates (β) of 5, 10, 15, and 20 °C min^–1^. The analysis was based on the Kissinger relationship:

ln­(β/*T*_p_^2^) = ln­(*AR*/*E*_a_)–(*E*_a_/*RT*_p_), where β is the heating rate and *T*_p_ is the peak temperature obtained from the DSC exotherm maximum.

Isothermal curing data were analyzed using apparent *n* ^th^-order fitting of the conversion profiles at constant temperature. Although epoxy–amine systems are fundamentally autocatalytic and are often better described by the Kamal or Sesták–Berggren formalisms, the present study intentionally employed simplified industrial DSC methodologies to critically evaluate their applicability and limitations for latent DGEBA–DICY curing systems.

The limitations of simplified *n* ^th^-order models for describing diffusion-controlled epoxy curing have been acknowledged. Advanced isoconversional approaches, such as the Friedman or Vyazovkin methods, would likely provide a more rigorous determination of the conversion-dependent activation energy and should be explored in future work.^35^

### Statistical Analysis and Assessment of Uncertainty

A single DSC experiment was conducted for each dynamic heating rate and each isothermal temperature. As a result, between-run repeatability and intermediate precision were not assessed, and no formal analysis of variance was carried out. For the Borchardt–Daniels calculations, the STARe software provided confidence limits for ln­(*Z*), activation energy, and reaction order. The reported ± values reflect the uncertainty of the numerical regression fit calculated by the STARe software. These values offer useful insight into the quality of the fit, but should not be considered equivalent to standard uncertainty, expanded measurement uncertainty, or experimental reproducibility.

For the Kissinger and isothermal Arrhenius analyses, the uncertainty was estimated using the standard error of the fitted regression slope. With four temperatures or heating rates available, the resulting confidence intervals have two degrees of freedom and should be interpreted with appropriate consideration.

Uncertainties related to sample mass and nitrogen flow were not incorporated into the confidence limits of the fitted kinetic parameters. While sample mass influences the mass-normalized enthalpy, its effect is largely negated in the Borchardt–Daniels conversion and reaction rate ratios. Uncertainties associated with purge-gas flow variations were not quantified in the present regression analysis.

## 3 Results and Discussion

### Dynamic DSC Analysis

The dynamic curing behavior of the DGEBA–DICY system was evaluated at heating rates (β) of 5, 10, 15, and 20 °C/min. The exothermic profiles (Figure 3) reveal a single reaction peak that shifts to higher temperatures with increasing heating rate, a characteristic feature of thermally activated kinetics. The reaction onset was observed at 170–190 °C, consistent with the known latency of micronized dicyandiamide, which requires elevated temperatures to dissolve and react with the oxirane rings.

**Figure 3:**
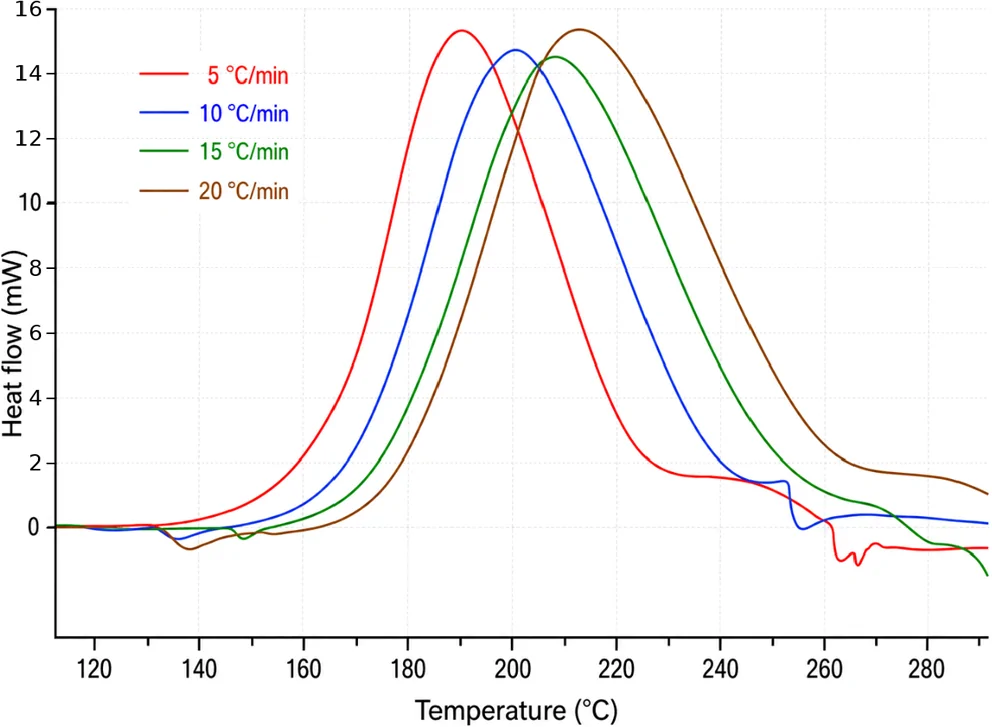
Dynamic DSC heat flow curves of the DGEBA–DICY system at heating rates of 5, 10, 15, and 20 °C min^–1^ under nitrogen atmosphere.

The integration of the exothermic peaks yielded total reaction enthalpies (Δ*H*) ranging from 118 to 124 J/g (Table 1), a spread of approximately 6% across the four heating programs. Because measurements were conducted across varying heating rates without replicates at each rate, this dispersion reflects method-condition variability rather than experimental repeatability.

**Table 1:** Borchardt–Daniels Apparent Kinetic Parameters and STARe Confidence Limits (CL)^a^

β		*E*_a_ ± CL	n ± CL	ln(Z) ± CL
(°C min^–1^)	(J g^–1^)	(kJ mol^–1^)	(−)	
5	117	176.93 ± 2.02	1.77 ± 0.02	40.79 ± 0.54
10	119	154.26 ± 3.39	1.75 ± 0.04	34.56 ± 0.89
15	124	138.01 ± 3.85	1.78 ± 0.05	30.04 ± 0.99
20	119	127.35 ± 4.18	1.70 ± 0.06	27.21 ± 1.06

a CL confidence limit reported by STARe software.

A small fluctuation observed near 260 °C occurs after completion of the main curing exotherm and is attributed to instrumental baseline instability at high temperature rather than to secondary chemical reactions or degradation phenomena. The absence of additional thermal events and the consistency of the measured reaction enthalpies support this interpretation.

Application of the Borchardt–Daniels model^2^ to the dynamic data yielded a reaction order (*n*) of approximately 1.7 across all heating rates. However, the calculated activation energy (*E*_a_) showed a strong dependence on the heating rate, decreasing from 177 kJ/mol at 5 °C/min to 127 kJ/mol at 20 °C/min.

The nonextruded mixture exhibited a reaction enthalpy of 89.45 J g^–1^ and a peak temperature of 234 °C, whereas the extruded material analyzed at the same heating rate exhibited 118.54 J g^–1^ and a peak temperature of 203 °C. Thus, extrusion increased the measured reaction enthalpy by approximately 32.5% and shifted the exothermic peak by approximately 30.7 °C toward lower temperatures. These results provide direct evidence that melt processing strongly modifies the thermal response of the formulation. However, DSC alone does not distinguish whether this modification results from improved dispersion, partial DICY dissolution, limited B-staging, or a combination of these effects.

This variation highlights a limitation in applying the Borchardt–Daniels method to complex curing systems. The Borchardt–Daniels model assumes a single-step *n*-th-order kinetic path (dα/d*t* = *k*(1−α)^*n*). However, epoxy-amine cures are intrinsically autocatalytic, because hydroxyl groups generated during epoxy ring opening accelerate subsequent reactions.

The observed shift in *E*_a_ suggests that the Borchardt–Daniels model imposes a phenomenological fit on a mechanism involving multiple competing steps (dissolution, chemically controlled reaction, and diffusion), the dominance of which changes with the heating rate.

The STARe confidence intervals for the activation energy do not overlap among the four heating rates. The differences among the fitted values are therefore substantially larger than the internal regression uncertainties. Nevertheless, because only one experiment was available at each heating rate, this observation does not establish statistical significance between the independent replicate measurements.

The variation in *E*_a_ indicates that the Borchardt–Daniels method provides a simplified phenomenological description of a curing process involving several physicochemical phenomena, including DICY dissolution, autocatalysis, network formation, and increasing restrictions on molecular mobility as curing proceeds. Therefore, the Arrhenius parameters obtained from the fits should be regarded as apparent or effective values rather than intrinsic chemical activation energies. Their dependence on heating rate is better understood as a consequence of changes in the relative contribution of these processes under different thermal histories, rather than as evidence of a change in the underlying curing mechanism. At lower heating rates, the material spends more time at intermediate conversions, giving mobility restrictions a greater influence on the measured reaction rate. At higher heating rates, in contrast, the response is more strongly dominated by the early stages of curing, when the reaction is mainly under chemical control.

The integrated reaction enthalpies ranged from 117 to 124 J g^–1^. Their mean was 120 J g^–1^, with a sample standard deviation of 3 J g^–1^ across the four heating programs. Because the measurements were obtained at different heating rates rather than as replicate runs, this dispersion should be interpreted as method-condition variability and not as repeatability uncertainty.

The activation energies obtained here are somewhat higher than those typically reported for conventional liquid DGEBA–amine systems (∼70–120 kJ/mol), likely reflecting the additional energetic contribution associated with DICY dissolution and diffusion limitations characteristic of latent-curing systems.^23–25^

To better understand how the reaction progresses and how the curing mechanism works, we calculated the degree of conversion (α) as a function of temperature. This was done by integrating the nonisothermal DSC exotherms according to α­(*T*) = Δ*H*_T_/Δ*H*_total_, where Δ*H*_T_ is the partial reaction heat released up to temperature *T* and Δ*H*_total_ is the total curing enthalpy. As shown in Figure 4, all the conversion curves display the typical S-shaped (sigmoidal) pattern and shift to higher temperatures as the heating rate increases, as expected for thermally activated reactions. The peak exothermic temperatures (*T*_p_) and their corresponding conversion values (α_p_) for each heating rate are listed in Table 2. Across all conditions, the maximum reaction rate (dα/d*t*)_max_, which matches the exothermic peak in the DSC data, happens at a moderate degree of conversion, between 34% and 39%. Traditional *n* ^th^-order kinetic models predict that the maximum rate should happen at the onset of the reaction (α → 0). In contrast, observing the maximum rate midway through the conversion process suggests that the DGEBA–DICY formulation cures via an autocatalytic mechanism. This finding is consistent with other studies on epoxy–amine curing reactions.^36,37^

**Figure 4:**
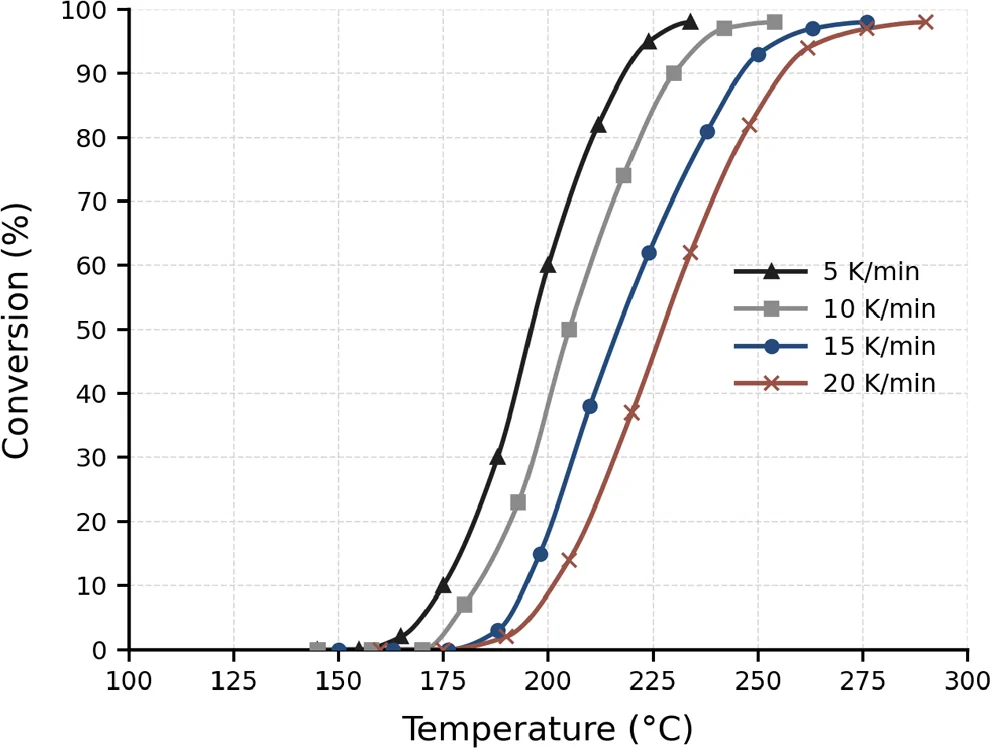
Degree of conversion (α) as a function of temperature for the DGEBA–DICY formulation at dynamic heating rates of 5, 10, 15, and 20 °C min^–1^.

**Table 2:** Exothermic Peak Temperatures (*T*_p_) and Degree of Conversion at Peak Maximum (α_p_) at Various Dynamic Heating Rates

β	Tp	α_p_
(°C min^–1^)	(°C)	(%)
**5**	∼190	∼36
**10**	∼201	∼39
**15**	∼208	∼37
**20**	∼213	∼34

### Peak-Based Multiple-Heating-Rate Kissinger Method

To mitigate the model dependence of the Borchardt–Daniels approach, the Kissinger method was applied. The peak-based Kissinger analysis was performed using four heating rates. Using the tabulated values and plotting −ln­(β/*T*_p_ ^2^) × 1000/*T*_p_ yielded a linear relationship (*R* ^2^ > 0.99). The slope corresponded to an apparent activation energy of approximately 108 kJ mol^–1^. With only four points, the slope has two degrees of freedom and an approximate 95% confidence interval of ±13 kJ mol^–1^. This interval describes regression uncertainty and not experimental reproducibility. The Kissinger value is a global peak-based estimate and should not be considered intrinsically more accurate than the Borchardt–Daniels results or interpreted as a conversion-resolved activation energy. This value lies between the extremes calculated by the Borchardt–Daniels method and likely represents an average of the energy barrier for the overall cross-linking process under dynamic conditions.

Unlike the Borchardt–Daniels analysis, the Kissinger method provides a global activation energy associated with the maximum reaction rate and does not capture how the kinetics change with conversion. This is important because, as discussed later in the residual-cure analysis (Figure 7), the apparent activation energy varies with conversion, suggesting that the relative contributions of chemical reaction and mobility restrictions change as the network develops. The Kissinger value should therefore be regarded as an apparent parameter describing the overall curing response under the applied dynamic conditions, rather than as the intrinsic activation energy of a specific reaction step. More advanced isoconversional methods, such as Friedman and Vyazovkin, could provide a more detailed evaluation of *E*_a_(α) and help distinguish chemical control from diffusion-related limitations. However, the present work was intentionally based on widely used industrial DSC methodologies.

### Isothermal DSC Analysis

Isothermal curing was conducted at 180, 190, 200, and 210 °C. A representative heat flow curve at 180 °C is shown in Figure 5, along with simulated curves based on the parameters of Borchardt–Daniels experiments with different heat rates.

**Figure 5:**
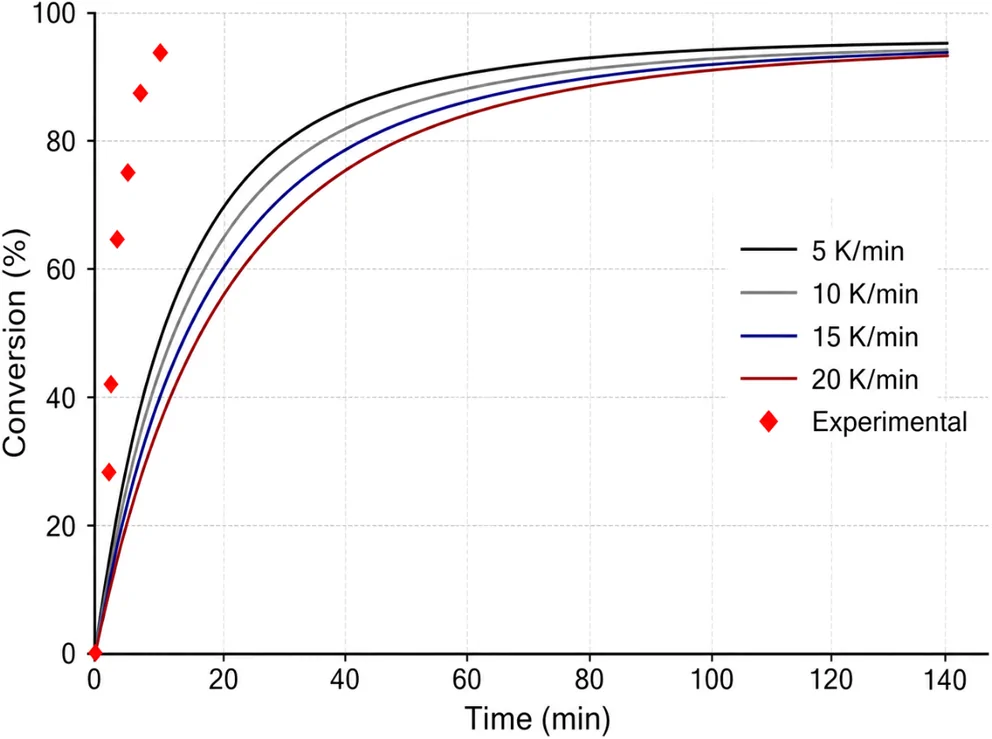
Experimental conversion at 180 °C obtained from residual-enthalpy measurements of separately precured samples, compared with conversion curves simulated using the Borchardt–Daniels parameters obtained at 5, 10, 15, and 20 °C min^–1^. Each experimental point represents one sample at one isothermal hold time; no replicate error bars were available.

### Analysis of the Instantaneous Peak

Contrary to typical autocatalytic epoxy-amine kinetics, which usually display an induction period corresponding to the generation of catalytic hydroxyls, the isothermal traces in this study exhibited a maximum reaction rate at approximately time zero, followed by a continuous decrease thereafter.

This behavior does not imply that the intrinsic autocatalytic curing mechanism has disappeared. Instead, it suggests that the induction stage was substantially suppressed because of the thermal history introduced during melt extrusion.

Two factors support this interpretation:

#### DICY Solubility

At temperatures equal to or above 180 °C, the dissolution rate of DICY is rapid. As reported in previous studies,^38,39^ when the curing temperature significantly exceeds the melting or dissolution threshold of the hardener, the induction period associated with phase homogenization becomes minimal, producing apparent pseudo-*n* ^th^-order kinetics despite the fundamentally autocatalytic nature of the reaction.

#### Thermal History (Extrusion)

The samples were prepared through melt extrusion. Melt extrusion at 90 °C combined thermal and shear history before DSC analysis. The nonextruded comparison confirms that processing modified the subsequent calorimetric response. Improved DICY dispersion, partial dissolution, and limited early-stage reaction are plausible contributors, but their individual contributions cannot be separated without complementary microscopy, XRD, FTIR, or chemical-conversion measurements. In addition, the finite sample-insertion time in the isothermal experiment may obscure an initial autocatalytic rise. The near-zero-time maximum should therefore be described as consistent with, rather than proof of, a suppressed induction stage.

The direct isothermal traces exhibited their maximum recorded heat flow near the beginning of data acquisition. This observation is compatible with a shortened induction stage in the processed material, but it may also be influenced by the sample-insertion and thermal-equilibration dead time. The DSC data do not distinguish these contributions. The fitted apparent reaction order increased from 0.73 to 1.00 over 180–210 °C (Table 3). The corresponding Arrhenius regression gave *E*_a_ ≈ 84 kJ mol^–1^ with an approximate 95% confidence interval of ±33 kJ mol^–1^ (two degrees of freedom). Consequently, the difference between the isothermal and Kissinger estimates is not statistically resolved by the available regressions.

**Table 3:** Apparent Kinetic Parameters Obtained from Direct Isothermal Fits^a^

*T*	Δ*H*	ln *k*(T)	Apparent *n*
(°C)	(J g^–1^)	(−)	(−)
180	118.42	–5.92	0.73
190	114.36	–5.28	0.83
200	122.85	–4.87	0.94
210	118.85	–4.53	1.00

a *k­(T)* is the apparent rate constant obtained from *n* ^th^-order fitting.

### Diffusion and Vitrification Effects

The residual-scan glass-transition temperature increased from approximately 50 °C at α = 0.28 to approximately 85 °C at high conversion. Even the maximum measured *T*_g_ remained about 95 °C below the isothermal curing temperature of 180 °C. Therefore, classical vitrification, defined by *T*_g_(α) approaching *T*_cure_, was not demonstrated under the present isothermal conditions. Modeling via the DiBenedetto equation was not evaluated due to the absence of unreacted (*T*_g0_) and fully cured (*T*_g∞_) baseline determinations. In a qualitative time–temperature–transformation interpretation, the 180–210 °C cure temperatures lie well above the measured *T*_g_ range. The late-stage kinetic changes are therefore discussed more cautiously in terms of gelation, increased cross-link density, and restricted molecular mobility, rather than vitrification.

### The Kinetic Compensation Effect

A comparison of the kinetic parameters reveals a discrepancy in the activation energies derived from the different methods: Isothermal (83.5 kJ/mol) < Kissinger (107.9 kJ/mol) < Borchardt–Daniels (127–177 kJ/mol). This numerical ordering should be interpreted cautiously. The approximate confidence intervals derived from the Kissinger and isothermal regression slopes overlap, and no experimental replicates were available. Thus, the results demonstrate method-dependent apparent activation energies but do not establish that all pairwise differences are statistically significant.

Despite these numerical differences, a Cremer–Constable plot of ln *A* versus *E*_a_ for all data sets (Figure 6) shows a strong linear correlation. This phenomenon, known as the kinetic compensation effect (KCE), has been widely reported in epoxy curing, solid-state decomposition, and heterogeneous catalysis.^29–33^

**Figure 6:**
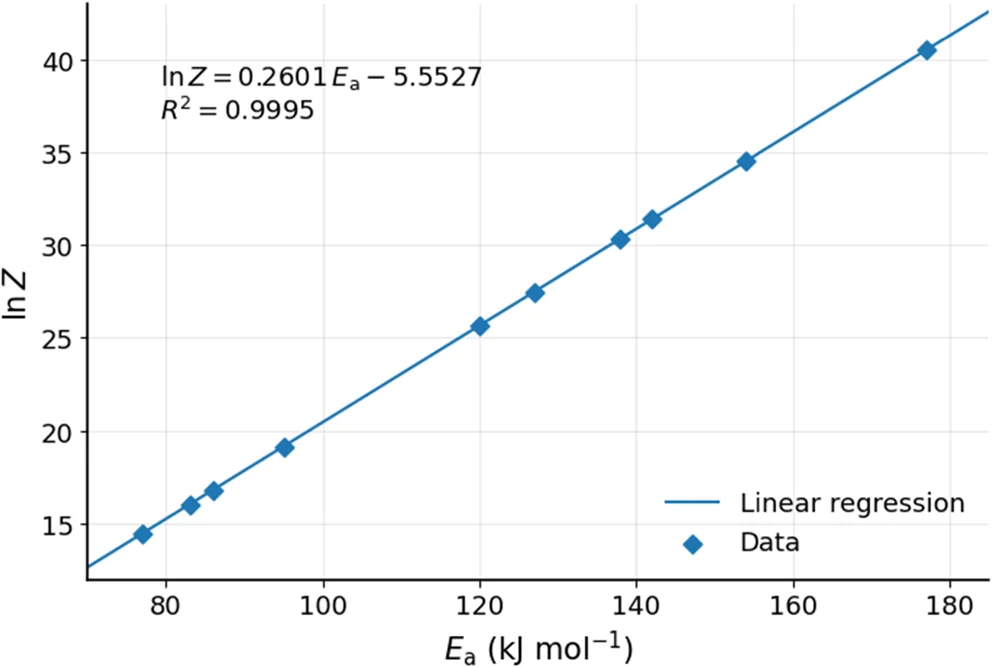
Cremer–Constable correlation between the fitted ln­(*A*) and *E*_a_ values obtained from the archived DGEBA–DICY kinetic analyses. The linear relationship indicates parameter correlation but does not by itself distinguish physicochemical compensation from regression covariance.

The DGEBA–DICY system exhibited substantial variation in *E*_a_ and ln­(*A*), which is consistent with kinetic heterogeneity in DICY curing and may be related to the chemically nonequivalent nitrogen-containing groups of DICY. However, the comparison does not isolate nitrogen reactivity from differences in hardener solubility, catalyst content, curing temperature, network formation, and reaction mechanism. Moreover, covariance between *E*_a_ and ln­(*A*) may generate an apparently linear Cremer–Constable relationship. Therefore, the participation of chemically distinct DICY reactive sites remains a plausible hypothesis rather than a demonstrated origin of the kinetic compensation effect.

However, the origin of the kinetic compensation effect (KCE) remains controversial. Several studies have shown that linear ln *A*–*E*_a_ relationships may arise not only from genuine physicochemical compensation but also from the mathematical covariance inherent in nonlinear Arrhenius fitting procedures.^29–33^ Therefore, the compensation effect observed here should not be interpreted as conclusive evidence of mechanistic invariance. Instead, it indicates a strong correlation between the Arrhenius parameters obtained using the different kinetic methodologies.

The Cremer–Constable plot shows a strong linear association between the fitted Arrhenius parameters. However, *E*_a_ and ln­(*A*) are estimated jointly and are intrinsically correlated in Arrhenius regressions. Because the covariance matrices of the original fits and independent replicate data sets were not available, the present data cannot distinguish genuine physicochemical compensation from statistical parameter covariance. Changes in hardener state, chemically nonequivalent DICY reactive sites, and network formation remain plausible physical contributors, but the linear relationship alone does not demonstrate their participation.

Figure 7 depicts the apparent Borchardt–Daniels activation energies obtained from the untreated 10 °C min^–1^ reference (α = 0) and from residual dynamic DSC scans of separate samples previously cured at 180 °C. Conversion was calculated from the residual reaction enthalpy. Each point represents one sample and one prior hold time. Point-specific fitting limits were not available for the residual scans; therefore, error bars are not shown.

**Figure 7:**
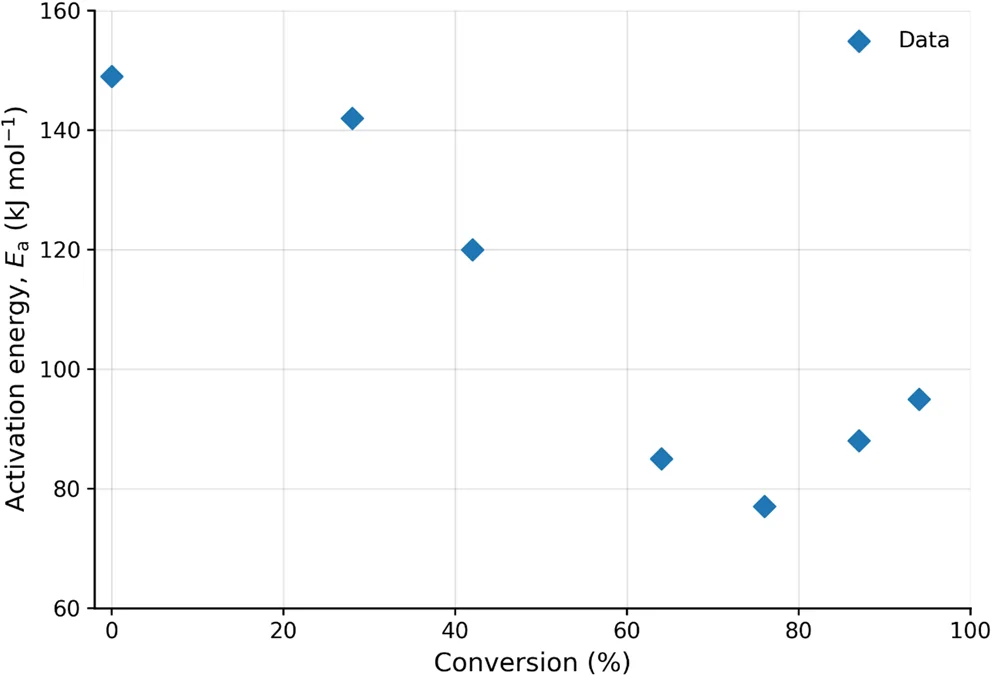
Apparent Borchardt–Daniels activation energies obtained from the untreated 10 °C min^–1^ reference (α = 0) and from residual dynamic DSC scans of separate samples previously cured at 180 °C.

The limited conversion series shows a marked decrease in apparent *E*_a_ up to α ≈ 0.75, followed by a modest increase at the last two measured conversions. Because each point was obtained from a different sample and no replicate or point-specific confidence limit was available, this nonmonotonic pattern should be regarded as exploratory. It is compatible with changing contributions from reaction chemistry and network-related mobility restrictions, but it does not establish a unique transition conversion or demonstrate vitrification.

## 4 Conclusions

This study shows that the kinetic parameters obtained for the industrial DGEBA–DICY powder formulation depend strongly on the DSC protocol and the kinetic model used. The Borchardt–Daniels analysis gave apparent activation energies that varied with heating rate, with differences much larger than the fitting limits reported by STARe. Since only one run was performed for each condition, however, experimental repeatability and statistical significance between conditions could not be assessed. The Kissinger and isothermal Arrhenius analyses gave lower overall estimates, with relatively broad and overlapping confidence intervals. Comparison with the nonextruded material indicates that melt processing changed the subsequent calorimetric response, although DSC alone cannot determine whether this was caused by improved DICY dispersion, partial dissolution, or some early-stage reaction.

The *T*_g_ increased with conversion but remained well below the isothermal curing temperature, so classical vitrification was not observed. The changes in *T*_g_ are more likely related to gelation, network formation, and the resulting restriction of molecular mobility. Similarly, the linear relationship between ln­(*A*) and *E*_a_ shows a strong correlation between the fitted parameters, but it does not establish whether this represents a real physicochemical compensation effect or simply a covariance between the Arrhenius parameters.

For routine industrial DSC measurements, Borchardt–Daniels parameters are therefore best treated as protocol-specific quality-control indicators. Meaningful comparisons require the sample preparation, processing history, storage conditions, sample mass, pan configuration, purge conditions, baseline treatment, integration limits, and heating rate to be kept consistent. Kissinger analysis can be useful for comparing the overall behavior of different samples, but neither approach should be considered a mechanistic model or used for extrapolation beyond the experimental conditions under which it was established.
